# Implementing health system and the new federalism in Somalia: challenges and opportunities

**DOI:** 10.3389/fpubh.2024.1205327

**Published:** 2024-02-01

**Authors:** Adam Sheikh Said, Dmitry Ivanovich Kicha

**Affiliations:** Department of Public Health, Healthcare and Hygiene, Peoples' Friendship University of Russia Named After Patrice Lumumba, Moscow, Russia

**Keywords:** health system, federalism, decentralization, health sector, challenges, opportunities, Somalia

## Abstract

In the 21st century, healthcare stands out as a formidable, contentious social responsibility for governments due to its high costs. This study delves into Somalia's healthcare system under Federal Government leadership, scrutinizing the complexities of health governance and financing. The Federal Government (FGS), along with governmental states (FMS) and regional authorities, collectively shoulder leadership and governance roles within Somalia's healthcare framework. Vital to resilient and inclusive development, the health sector holds a pivotal role. A strategic investment in healthcare not only drives substantial demographic dividends through enhanced life expectancy and reduced fertility rates, but also paves Somalia's trajectory toward progress. The Federal Government of Somalia confronts a multitude of challenges in its pursuit of effective healthcare implementation. A prominent obstacle lies in health financing. Somalia relies heavily on international and private sources for health support, primarily due to limited government revenue generation. This financial shortfall restrains the government's capacity to allocate ample funds for public services and critical investments, including healthcare. This paper sheds light on the present healthcare landscape in Somalia and expounds on the hurdles confronted by healthcare systems under federal governance. Moreover, it delves into the historical evolution of Somalia's healthcare system and the advent of new federalist principles. In doing so, this study comprehensively examines the dynamics of healthcare governance, financing, and historical progression in Somalia.

## Introduction

The term “federalism” denovtes the mutual agreement among multiple political entities to establish a foundational framework for a union. This concept encompasses a sequence of independent agreements between different nations, enabling the sharing of federal institutions while preserving a measure of their individual autonomy (1). Somalia's adoption of federalism dates back to 2004, but the concrete execution of federalism within Somalia commenced in 2012 when Hassan Sheik and his Ministries established a regional state comprising significant regions, namely Galmudug, Hirshabeelle, Southwest, and Jubaland (2).

The enduring humanitarian crisis spanning over two decades in Somalia has inflicted substantial harm upon both developmental progress and public health. The endeavor to implement these federalist structures remains a complex issue, and the protracted civil war has taken a severe toll on the health of the Somali populace. Armed conflicts have ravaged healthcare infrastructure, resulting in limited access to vital health services. This dire situation has exposed an already vulnerable demographic to heightened disease prevalence and malnutrition (3).

This study strives to offer essential insights into how Somalia's healthcare system has been received and operated amidst the introduction of the federal governmental framework. It delves into the ongoing restructuring of the country's health system, offering a synthesis of the advancements, obstacles, and prospective strategies for addressing healthcare challenges. By scrutinizing the role of the new federal government in redefining healthcare administration, the research will underscore various encountered hurdles. Specifically, it will spotlight significant practical obstacles impeding Somalia's federal healthcare delivery, encompassing challenges in both provisioning and accessibility of healthcare services. These include the privatization and urban-centric focus of the existing healthcare system, which leaves rural populations devoid of affordable healthcare access. Furthermore, the decentralization of the federal health system and the absence of cohesive management hinder national authorities from effectively overseeing the private sector and partnering with non-governmental organizations to extend care to rural areas. These challenges, compounded by a lack of public comprehension regarding the health implications of federalism, are steering the nation toward a decentralized healthcare system.

Drawing on experiences and insights from other nations that have implemented federalism, the paper will highlight key considerations for managing health sector federalism.

## Current situation of health system in Somalia

**Figure 1:**
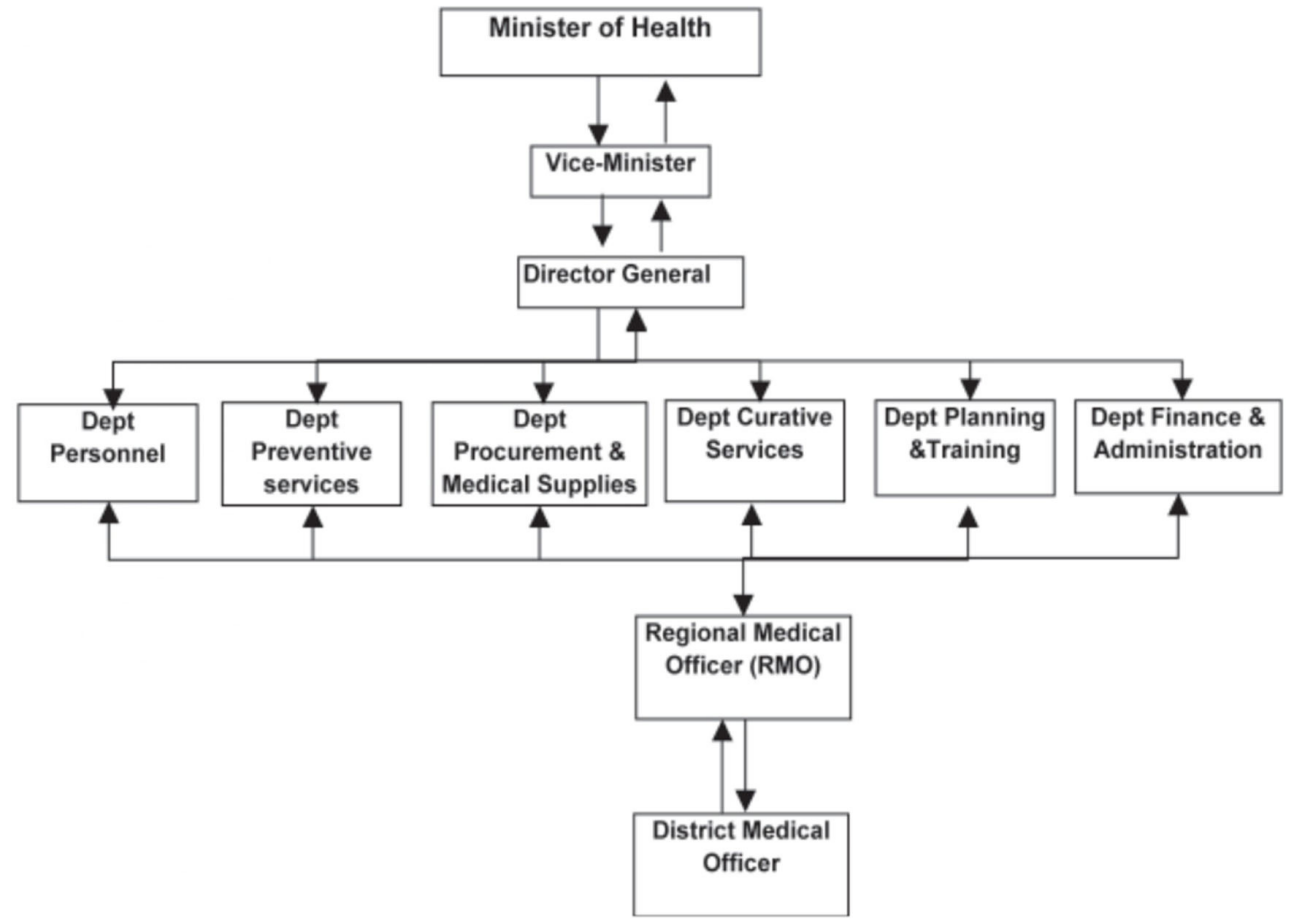
Structure of the healthcare system of Somalia. Source: Qayad (5).

**Figure 2:**
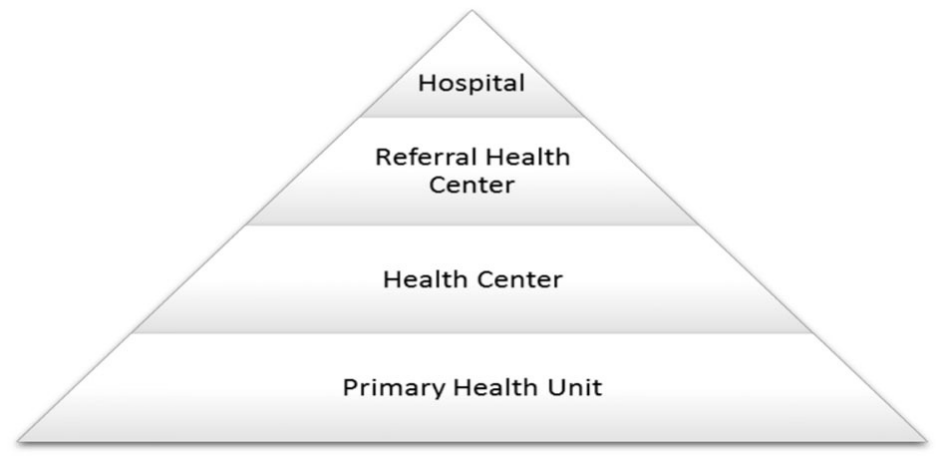
Current Somali health system. Source: Health (6).

In the 21st century, the weighty responsibility and contentious nature of healthcare as a government social policy are evident (3). In Somalia, about 846 healthcare facilities exist, with 7 reference hospitals, 27 district hospitals, 248 maternity and child health clinics, and 544 health posts (4). The MOH uses a centralized decision-making system and has linkages to the 18 administrative regions via regional medical officers (RMO), who report to the curative services department (Figure 1) (5). The Somali public healthcare system is organized into four levels, including Primary Health Units (PHUs) in rural areas, Health Centers (HCs) at the sub-district level, Referral Health Centers (RFCs) in districts, and Regional Hospitals (RHs) located in the regional capitals (Figure 2) (7). The healthcare system grapples with significant challenges in meeting the diverse health needs of its population. With an estimated population of 12.3 million, the country comprises a mix of urban, rural, nomadic, and internally displaced individuals (3). Life expectancy in Somalia remains relatively low, reflecting a substantial burden of diseases such as malaria, cholera, and malnutrition, compounded by the health impact of long-standing conflict. Trauma-related injuries and mental health issues also contribute to the complex healthcare landscape (8). Despite these challenges, the expectations of the population for accessible and high-quality healthcare services are substantial. Communities play an essential role in addressing health needs, often relying on traditional healers and community health workers due to the scarcity of formal healthcare facilities (9).

Healthcare financing in Somalia presents several complex issues. The country's healthcare landscape features a mix of private providers dominating the sector, and the government's expenditure on healthcare is limited. Consequently, a significant proportion of healthcare costs is covered through out-of-pocket payments by individuals. International comparisons reveal that per capita expenditure on healthcare in Somalia is notably low (10). To bridge funding gaps and enhance access to healthcare services, Somalia relies on donor aid and development projects, such as the “Damal Caafimaad Project” funded by the World Bank (11). However, the reliance on donor aid and out-of-pocket payments raises concerns about the sustainability and equity of the healthcare financing system.

The healthcare workforce in Somalia is unevenly distributed, with a heavy focus on urban areas, leaving rural and remote communities underserved. The protracted conflict has displaced over 2.2 million internally displaced persons (IDPs), and around 600 medical professionals have migrated, leading to urban-rural and public-private imbalances in the healthcare workforce (12). Critical shortages of qualified healthcare staff hinder Somalia's federal health services. Nurse, midwife, and doctor densities below 4 per 10,000 individuals (far from the required 23) point to a shortage of around 24,000 workers to meet minimum thresholds, particularly acute in remote areas (13).

United Nations' perspective envisions independent regional states in health service delivery, with federal states coordinating actors within regions (NGOs, UN agencies, and private providers). However, communication between tiers remains suboptimal, leading to activities endorsed by federal states without proper state-level communication (14). Service delivery in Somalia is characterized by an urban-centric approach, leading to disparities in access to healthcare services. The primary care system plays a crucial role in delivering essential healthcare services, but its reach remains limited in many parts of the country. Public health services face challenges in reaching underserved populations, including internally displaced individuals (15).

The governance of Somalia's healthcare system is complex, involving multiple layers of government and stakeholders. The Federal Ministry of Health and Human Resources (FMoH) is responsible for quality control, policy formulation, human resource development, and coordination. However, capacity constraints and the unclear federal system have hindered FMoH's nationwide jurisdiction. Coordination and communication between different tiers of government and stakeholders, including NGOs, UN agencies, and private providers, have been suboptimal, leading to challenges in the implementation of health policies and programs (16). The establishment of the National Health Professions Commission (NHPC) by the Federal Ministry of Health exemplifies the federal government's pivotal role in regulatory matters, particularly in safeguarding against medical malpractice and promoting ethical conduct within the healthcare sector (17, 18).

## Challenges faced by the federal government in healthcare implementation and delivery

Federalism in Somalia has profound implications for the governance and delivery of healthcare services, which are essential for the wellbeing of the population. The country operates as a federal state, composed of various regions and states, each wielding varying degrees of autonomy over healthcare management and decision-making. This decentralization of power carries several implications and challenges for the healthcare system (19).

One critical consequence of federal system is the fragmentation of healthcare services. With each region and state having the authority to formulate its healthcare policies and regulations, uniformity and standardization of healthcare practices across the nation become elusive. Consequently, patients may experience disparities in the quality and accessibility of healthcare services, depending on their geographic location. These regional discrepancies can erode public trust and hinder equitable access to quality healthcare services, which is critical for a healthy and prosperous society (11).

Resource allocation disparities also emerge as a significant concern within the framework of federalism. Regions with more abundant resources and robust governance structures may have the capacity to develop better healthcare infrastructure and services. In contrast, less-developed regions may face formidable challenges in delivering even the most fundamental healthcare services to their populations. This inequality in resource allocation underscores the pressing need for a coordinated and equitable approach to healthcare financing to ensure that all citizens, regardless of their geographic location, have access to essential healthcare services (20).

Coordination challenges arise from the complex interplay between the federal government and regional authorities in healthcare management. Competing interests, disputes over authority, resource allocation, and policy direction can hamper cooperation and undermine the overall delivery of healthcare services. Ensuring that all regions work cohesively within a shared healthcare framework is critical for the effective functioning of the healthcare system (21).

Furthermore, regional disparities in healthcare policies and regulations can lead to inconsistencies in the healthcare system. Divergent policies can result in a lack of a coherent national healthcare strategy, impeding the implementation of comprehensive and well-integrated healthcare systems at the national level. In light of this, the establishment of unified national healthcare policies that encompass the unique needs and circumstances of each region becomes an imperative (22).

Federalism also brings challenges in resource mobilization and funding for the healthcare system. The federal government may encounter difficulties in mobilizing resources and securing funding, particularly given that regional governments may have their revenue sources and differing priorities. Hence, a coordinated approach to healthcare financing that bridges regional and federal initiatives is necessary to provide a reliable source of funding for healthcare services (13).

The capacity of regional healthcare authorities and the effectiveness of governance structures at both the federal and regional levels are pivotal for a functional healthcare system. To address this, investment in capacity building and training programs for healthcare professionals and administrators at both federal and regional levels is needed to enhance the quality of healthcare services. Furthermore, strengthening regulatory oversight is imperative to ensure that healthcare services meet quality and safety standards across regions, regardless of their autonomy level (23).

### Brief history of healthcare system in Somalia

Healthcare system in Somalia has changed and evolved significantly over the years (see Table 1). The table offers a chronological summary of significant historical milestones in the health care system development that include Somalia's challenges and recovery attempts over time. This historical perspective is crucial to understanding the present condition of healthcare in Somalia and its persistence through all those misfortunes.

**Table 1:** Brief history of healthcare system in Somalia.

**References**	**Year**	**Events**
Bile et al. (24)	July 1, 1960	Somalia inherited a weakened post-colonial health-care system focused on curing rather than preventing disease, with most rural and nomadic populations having little access to essential health-care services. The expansion of health facilities and mobile outreach services over the next three decades resulted in significant improvements.
WHO (25)	1970s	An effort was made in the 1970s to encourage the building of healthcare facilities and to increase the number of other health professionals. In order to accomplish this, two nursing schools were established, along with numerous other health-related educational initiatives. Equally significant was the dispersal of medical professionals and resources across the nation. The majority of workers and facilities were centered on Mogadishu and a few other towns at the beginning of the 1970s. By the late 1970s, things had considerably changed, but the distribution of medical treatment remained inadequate.
Qayad (5)	1970s	Academic institutions improved the quality of the training they provided to healthcare professionals, who were then used throughout the healthcare system. Many prominent Somali doctors pursued postgraduate studies at Italian universities during the same time period, and upon their return, they were involved in academic teaching by instructing medical students, nurses, midwives, and a variety of other health professionals.
Global Health Action (26)	1980s	Through the National Academy of Science and Arts in Mogadishu, the faculty of medicine launched research in the medical sciences in conjunction with numerous Swedish universities. In Somalia, research in the medical sciences and other sectors had a new birth at this time. This program and the others described before primarily aided in the development of health-related manpower.
Global Health Action (26)	Early, 1990s	Government spending on health declined gradually, from 4 to 5% of total spending in the 1970s and early 1980s to only 2%. Access to health care had deteriorated even further, with only Mogadishu and areas supported by the international community providing some services. It was estimated that 80% of the population lacked basic health care. As expected, 15 years of conflict have had a significant impact on the health system, affecting all of its components: human resources, infrastructure, management, service delivery, and support systems.
World factbook (27)	1991	The civil war in this year led ton destruction of the modest health infrastructure of the country. Its premises were vandalized, looted and taken over by poor squatters, internally displaced people and at times armed tribal militias. The conflicts also caused internal displacement of more than 1,000 health workers to safer urban centers, while another 600 doctors, nurses, and midwives, constituting about 20% of the workforce, migrated during the first five years of the civil war. This disruption was also accompanied by the closure of the only medical school in the country.
Dalmar et al. (28)	Early 2010s	The transition to recovery of health-system organization, regulation, and workforce development began with the establishment of a transitional federal government, which created opportunities to begin the pursuit of universal health coverage (UHC). During this recovery period, approximately 25 academic institutions with undergraduate medical or health courses and a few master's training courses were operationalized, and steps were taken to re-establish collaboration with Swedish universities.
Warsame et al. (3)	2013	Since the New Deal Somalia Conference in Brussels in 2013, the country has entered a new phase of political and economic development, with the Federal Government and the international community signing a Compact for sustainable peace, increased service provision, and economic development. Somali leadership was seen as crucial in areas such as health system strengthening. This has taken the form of regional health sector strategic plans being drafted by respective ministries with ongoing support from UN agencies. These plans are currently being implemented through the multi-donor funded Joint Health and Nutrition Program (JHNP), in which the state is contracting out health services to implementing partners under the technical assistance program by UN agencies.
Ahmed et al. (29)	2018	Poor environmental conditions, limited access to water, and insufficient sanitation facilities—exacerbated by flooding and Cyclone Sagar—added to the overwhelming impact of the 2017 drought, driving up levels of malnutrition and disease across the country. Access to healthcare continues to deteriorate as a result of widespread, persistent violence, and the health system remains fragmented, under-resourced, and ill-equipped to provide lifesaving and preventative services.

### Healthcare systems and federalism in other countries: a lesson for Somalia?

In the 1990s, certain nations, such as Ethiopia (1995), South Africa (1996), and Nigeria (1999), exhibited fledgling federal structures, with some having deeper historical backgrounds of failed federalism. Ethiopia's experience encompassed the imperial era and the military dictatorship (1974–1991), transitioning from a centralized nation to a parliamentary-federal Constitution due to a struggle by ethnolinguistic groups (30). Notably, global conflicts, like those witnessed by Somalia, were not unique; 54 wars occurred in 2019, many now in post-conflict phases, shifting from open violence. Countries like Côte d'Ivoire, Cameroon, Burkina Faso, Chad, Niger, Mali, and Nigeria in Africa faced armed conflicts (31). These wars have been demonstrated to adversely affect health systems and public health. Thus, Somalia's post-conflict period offers an exceptional opportunity for healthcare system reform (32), aiming to rectify pre-war health system deficits, war-induced public health ramifications, and healthcare system strains.

Despite these challenges, other countries have rebounded from war, establishing robust healthcare systems. Somalia can draw insights from these cases for healthcare and federal government structure reform. Ivory Coast, recovering from civil war, stands as a relevant model. Ivory Coast sought quality healthcare during the 1960s−1980s, funded entirely by the state. A shift toward tertiary healthcare in urban centers, exemplified by constructing a university hospital in Abidjan, marked this period (33). The WHO's engagement countered urban healthcare favoritism, aiming for comprehensive coverage. Amidst a civil war ignited by the 2010 elections, Ouattara emerged victorious but encountered post-conflict healthcare disruption (34). Medical professionals fled, facilities shuttered due to conflict, and over 70% lacked healthcare access by 2011 (33). Despite these challenges, Ivorian healthcare recovery initiatives included universal healthcare and a new National Health Development Plan (NHDP). Challenges remain due to financial and political hurdles (33).

In the context of the Ivory Coast case study, Somalia can draw valuable lessons from its own positive outcomes, particularly in regions like Somaliland. These areas have already made significant progress in terms of improved security and governance, which has led to the establishment of more functional healthcare systems. For example, Somaliland has seen advancements in healthcare infrastructure and an increase in the availability of trained medical professionals (35). These positive outcomes within Somalia's own borders provide a compelling example of how improvements in security and governance can positively impact healthcare delivery. By building on these local successes, Somalia can foster similar healthcare recovery initiatives and apply the lessons learned to develop tailored healthcare policies and reforms that address both immediate needs and longstanding obstacles, aligning with the progress made in regions like Somaliland.

## Recommendations and future prospects

The enhancement of public health infrastructure is paramount, as it not only amplifies accessibility but also augments the efficiency and caliber of services. Federalism continues to offer promising avenues for Somalia's health system advancement. The proximity of local governance to the populace underlines federalism's potential to bolster health resources, funding, and services for the people. While foreign entities, donors, and the United Nations system have extended substantial aid to primary care facilities across Somalia, these efforts have proven inadequate, necessitating further assistance, including the establishment of new facilities and the restoration and expansion of existing ones, encompassing vital components like blood banks and laboratory amenities. Mapping capacity development and formulating policies to align with structural and functional changes, including institutional arrangements, becomes imperative. Consequently, the federal government's urgent task involves the training of fresh cohorts of healthcare personnel (18, 36). The harmonization of educational and curriculum approaches across public health training institutions is equally pressing to elevate training quality and produce adept medical professionals equipped with essential knowledge and skills.

To ensure the effectiveness of newly constructed healthcare infrastructure, conducting a comprehensive situational analysis is vital. This analysis helps assess healthcare needs and requirements, ultimately preventing issues like underutilization or overcrowding of healthcare services. The assessment should take into account medical necessities and local concerns, serving as the foundation for the development of short, mid-term, and long-term strategic plans aimed at revitalizing the nation's healthcare system (32, 37).

Accommodating the unique requirements of Somalia necessitates specialized implementation strategies, encompassing uniform health records and resilient, effective medicine delivery systems, alongside infrastructure expansion to meet the nation's health exigencies (37).

In post-conflict Somalia, the pivotal engagement of the local business community is essential, even though their contributions often come in sporadic and fragmented forms. To address issues of fragmented healthcare systems and promote a more effective approach to healthcare service delivery, the government must establish early-stage coordination mechanisms during the recovery phase. Furthermore, the ongoing capacity-building process should focus on various levels, including contextual, systemic, organizational, and individual elements, with the support of operational tools designed to reinforce the development of crucial leadership skills. This comprehensive approach not only prevents uncontrolled healthcare service growth but also enhances the necessary managerial capabilities within local communities and public sector entities, ensuring a more sustainable and efficient healthcare system (32, 37).

The expansion of healthcare services or the inception of new projects necessitates close synergy between the private and public sectors, an imperative that was exacerbated during periods of conflict. Streamlined, practical, and concise collaborative strategies tailored to address the nation's healthcare needs offer a superior alternative, as they are less reliant on donors, economically sustainable, and adaptable when donor agencies phase out or reduce funding (32, 37).

## Conclusions

Federalism presents a provides a platform for necessary work and reforms for the nation to achieve Universal Health Coverage (UHC), contingent upon various critical factors such as legislative backing, robust funding mechanisms, quality standards, capacity enhancement, and effective governance structures. The study's insights accentuate the importance of regulatory frameworks for equitable and affordable private healthcare services, a pressing need in Somalia. Additionally, the emphasis on patient-provider interactions within the private healthcare sector underscores the significance of patient care improvements. To ensure a well-rounded approach, it is imperative to consider the interplay between federalism and decentralization in shaping the regulatory environment, promoting patient-centered care, and enhancing medical education curricula. By harnessing the strengths of federalism while incorporating the benefits of decentralization, Somalia's healthcare system can progress toward greater inclusivity, improved patient outcomes, and enhanced healthcare professionalism.

## Author's note

AS (MD, Ph.D.) is an ENT doctor head and neck surgery and a public health expert specializing in health systems. Their PhD thesis focused on the health systems in Somalia at RUDN University. During time in Somalia, they served in major hospitals and published research papers on the Health System in Somalia and were one of the board Staff Health Institutions in Somalia. Their expertise in health systems has allowed them to provide medical care to those in need in Somalia and to understand the complexities of health system reform in a developing country. They are committed to improving health systems in developing countries and to continuing research on health systems in Somalia. DK is an honored Professor of RUDN University, Doctor of Medical Sciences, Academician of the Russian Academy of Medical and Technical Sciences, Deputy Chairman of the RAMS Problem Commission on Public Health, member of the Expert Council of the Higher Attestation Commission of the Russian Federation on Medical and Preventive Sciences, member of the RUDN University Certification Commission for the Award of Scientists degrees, member of the editorial boards of journals, advisor to the chief physician of the Center for Hygiene and Epidemiology in Moscow, chairman of the educational committee of the FNMO MI RUDN University and expert in the health systems of low income countries. In 1987–1989 they were an adviser to the World Health Organization on methodology for training medical personnel in the WHO Pacific Region-Manila, Philippines (1987–1989).

## Data availability statement

The original contributions presented in the study are included in the article/Supplementary material, further inquiries can be directed to the corresponding author.

## Author contributions

All authors listed have made a substantial, direct, and intellectual contribution to the work and approved it for publication.
